# KCNK3 inhibits proliferation and glucose metabolism of lung adenocarcinoma via activation of AMPK-TXNIP pathway

**DOI:** 10.1038/s41420-022-01152-9

**Published:** 2022-08-13

**Authors:** Guofu Lin, Lanlan Lin, Hai Lin, Wenhan Chen, Luyang Chen, Xiaohui Chen, Shaohua Chen, Qinhui Lin, Yuan Xu, Yiming Zeng

**Affiliations:** 1 Department of Pulmonary and Critical Care Medicine, The Second Affiliated Hospital of Fujian Medical University, Quanzhou, Fujian province 362000 China; 2 Respiratory Medicine Center of Fujian Province, Quanzhou, Fujian province 362000 China; 3 The Second Clinical College, Fujian Medical University, Quanzhou, Fujian province 362000 China; 4 Department of Pathology, The Second Affiliated Hospital of Fujian Medical University, Quanzhou, Fujian province 362000 China; 5 Clinical Research Unit, The Second Affiliated Hospital of Fujian Medical University, Quanzhou, Fujian province 362000 China

**Keywords:** Non-small-cell lung cancer, Cell signalling

## Abstract

Non-small cell lung cancer (NSCLC) is a primary histological subtype of lung cancer with increased morbidity and mortality. K^+^ channels have been revealed to be involved in carcinogenesis in various malignant tumors. However, TWIK-related acid-sensitive potassium channel 1 (TASK-1, also called KCNK3), a genetic member of K2P channels, remains an enigma in lung adenocarcinoma (LUAD). Herein, we investigated the pathological process of KCNK3 in proliferation and glucose metabolism of LUAD. The expressions of KCNK3 in LUAD tissues and corresponding adjacent tissues were identified by RNA sequencing, quantitative real-time polymerase chain reaction, western blot, and immunohistochemistry. Gain and loss-of-function assays were performed to estimate the role of KCNK3 in proliferation and glucose metabolism of LUAD. Additionally, energy metabolites of LUAD cells were identified by targeted metabolomics analysis. The expressions of metabolic molecules and active biomarkers associated with AMPK-TXNIP signaling pathway were detected via western blot and immunofluorescence. KCNK3 was significantly downregulated in LUAD tissues and correlated with patients’ poor prognosis. Overexpression of KCNK3 largely regulated the process of oncogenesis and glycometabolism in LUAD in vitro and in vivo. Mechanistic studies found that KCNK3-mediated differential metabolites were mainly enriched in AMPK signaling pathway. Furthermore, rescue experiments demonstrated that KCNK3 suppressed proliferation and glucose metabolism via activation of the AMPK-TXNIP pathway in LUAD cells. In summary, our research highlighted an emerging role of KCNK3 in the proliferative activity and glycometabolism of LUAD, suggesting that KCNK3 may be an optimal predictor for prognosis and a potential therapeutic target of LUAD.

## Introduction

Lung cancer is one of the predominant malignant tumors with a considerable morbidity and mortality rate in both sexes worldwide, accounting for 11.4% of total cancer cases in 2020 [1]. Lung adenocarcinoma (LUAD) comprises ~80% of lung cancer cases [2]. Due to the lack of effective therapeutic strategies, the average five-year survival rate of LUAD patients is only 15% [3, 4]. Surgical resection is the fundamental first-line treatment modality for operable stage I LUAD. While disease recurrence remains a vexing problem for this population [5]. Therefore, it is imperative to identify postoperative early-stage LUAD patients at high risk of loco-regional recurrence to assist with adjuvant therapy.

K^+^ channels are transmembrane proteins that selectively regulate the flow of K^+^ along an electrochemical gradient [6]. These molecules were found to participate in a variety of tumorous process, including cellular proliferation, migration, invasion, and apoptosis [7, 8]. Two-pore domain K^+^ channels (K2P channels), as one of the main classes of K^+^ channels, can be classified into four different classes, such as weak inward rectifiers and acid-sensitive K^+^ channels (TASK-1 and TASK-2) [9]. TWIK-related acid-sensitive potassium channel 1 (TASK-1, also called KCNK3), an essential member of the K2P channels, is sensitive to various environmental and physiological conditions that may influence their bioactivities, such as hypoxia, unsaturated fatty acids, acidic level, and intracellular signaling pathways [10, 11]. Previous studies have identified that KCNK3 was functionally expressed in pulmonary arterial smooth muscle cells, intestine, heart, and bladder [11, 12]. Moreover, KCNK3 has been proved to be related to multiple kinds of cancer including neuroblastoma [13], pancreatic cancer [14], colorectal cancer [15], and hepatocellular carcinoma [16].

Although the effects of KCNK3 on NSCLC have been reported, its anti-cancer potential and underlying mechanism on LUAD have not yet been investigated. For instance, Leithner K et al. showed that KCNK3 was expressed in NSCLC cell lines but not altered in cancerous tissues when compared with normal pulmonary tissues, and KCNK3 was found to be able to regulate apoptosis in NSCLC cell lines [17]. However, its additional biological role and profound mechanism of KCNK3 in early-stage lung cancer, especially adenocarcinoma, are still unknown.

In the current research, we investigated the clinical relevance and potential effects of KCNK3 in LUAD. We demonstrated that KCNK3 was significantly downregulated in stage I LUAD tissues based on bioinformatic analysis and experimental validation. Subsequent in vitro and in vivo assays indicated KCNK3 modulated the proliferation and glucose metabolism of LUAD cells via activation of AMPK pathway. Our findings implicated that KCNK3 could be served as a key molecular regulator of cell metabolism and aberrant cell proliferation of LUAD, which provides a further understanding of the underlying pathogenesis of lung cancer.

## Results

### Decreased expression of KCNK3 correlated with poor prognosis of stage I LUAD patients

**Fig. 1:**
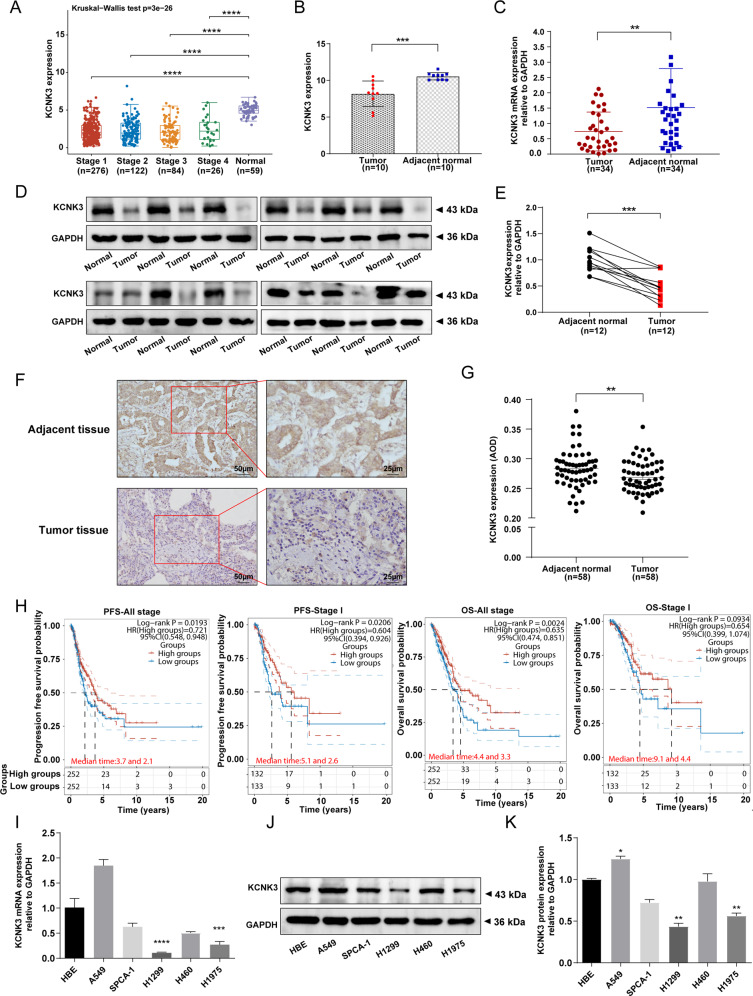
Low KCNK3 expression in LUAD tissues was correlated with poor prognosis. **A** The KCNK3 mRNA levels in LUAD tissues compared with the normal lung tissues from TCGA database. **B**, **C** The mRNA expression of KCNK3 in stage I LUAD patients was determined by RNA sequencing (*n* = 10) and qRT-PCR (*n* = 34). **D**, **E** Western blot analysis of KCNK3 protein expression in stage I LUAD tissues and corresponding adjacent tissues, with results normalized relative to the expression of GAPDH (*n* = 12). **F**, **G** KCNK3 protein expression in stage I LUAD tissues and adjacent tissues were detected by IHC and quantitative analysis by using average optical density (AOD, AOD = IOD/area, *n* = 58, scale bar 50 or 25 µm). **H** The prognostic values of KCNK3 in LUAD patients from TCGA by using a clinical bioinformatics database (www.aclbi.com). **I**–**K** KCNK3 mRNA and protein expression levels of LUAD cell lines were assessed by qRT-PCR and western blot, respectively. (OS, overall survival; PFS, progression-free survival. *P < 0.05, **P < 0.01, ***P < 0.001, ****P < 0.0001).

To investigate the expression of KCNK3 in LUAD, we first identified gene expression profiles of KCNK3 between cancer and normal tissues through a database-mining approach. In the 508 primary LUAD tissues and 59 normal samples originated from the Cancer Genome Atlas (TCGA), we found that KCNK3 was significantly downregulated in different clinical stages of LUAD tissues compared with non-tumor tissues, especially in stage I LUAD patients (Fig. 1A). Subsequently, we performed RNA sequencing to detect genic expression profile in 10 pairs of stage I LUAD and corresponding adjacent tissues. Our results also presented that the expression of KCNK3 was remarkably reduced in stage I LUAD tissues (Fig. 1B). Furthermore, we conducted quantitative real-time polymerase chain reaction (qRT-PCR) analysis to validate TCGA data and RNA-seq results. The qRT-PCR data suggested that KCNK3 mRNA expression level was markedly lower in LUAD than in adjacent tissues (Fig. 1C, *n* = 34). We then detected the KCNK3 protein expression in 12 paired LUAD and adjacent tissues by western blot, the result indicated that KCNK3 was markedly downregulated in stage I LUAD patients (Fig. 1D, E). Immunohistochemical staining was further conducted to confirm the expression of KCNK3 in LUAD tissues (Fig. 1F). As presented in Fig. 1G, the average optical density (AOD) value of immunohistochemistry was substantially elevated in adjacent tissues (Fig. 1E, *n* = 58).

Moreover, we analyzed its prognostic implication of KCNK3 in LUAD from TCGA databases based on a clinical bioinformatics database (www.aclbi.com). Kaplan-Meier survival curve showed that downregulated expression of KCNK3 exhibited a remarkedly worse progression-free survival (PFS) in all stage LUAD patients. A similar tendency of overall survival (OS) was also observed except for stage I LUAD patients (Fig. 1H).

Additionally, we assessed the expression of KCNK3 in LUAD cell lines and normal bronchial epithelial cell line HBE. The results showed that KCNK3 mRNA and protein expressions were downregulated in LUAD cells, especially in H1299 and H1975 cells (Fig. 1I–K).

### Aberrant KCNK3 expression modulated the proliferation of LUAD cell lines

**Fig. 2:**
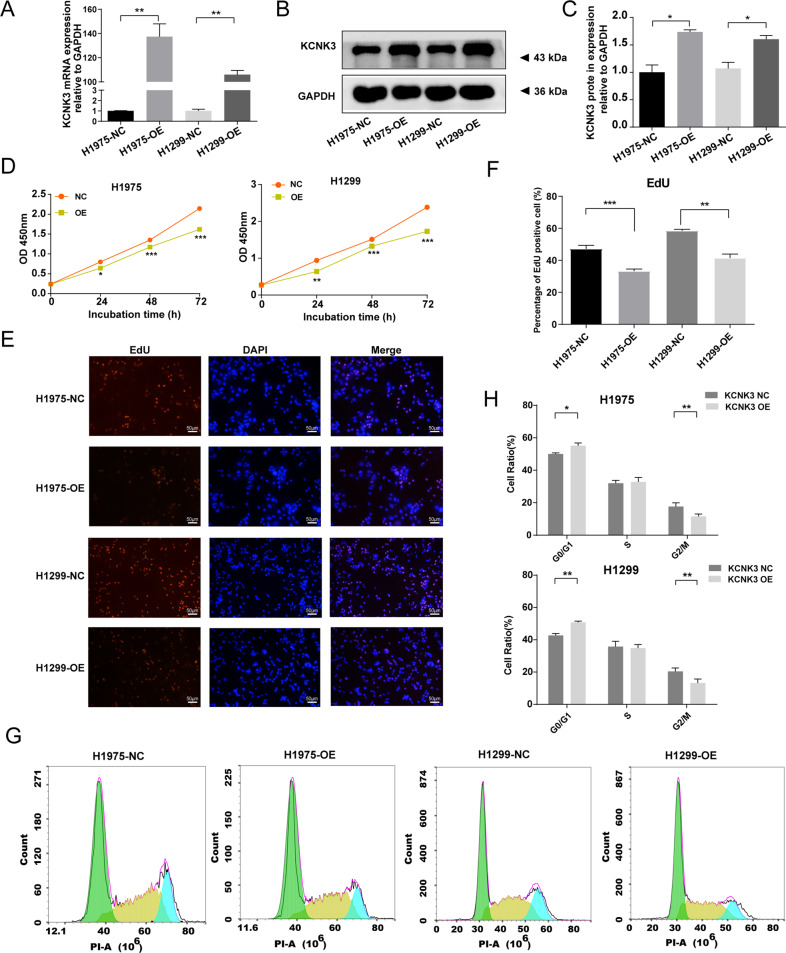
Upregulation of KCNK3 expression inhibited the proliferation of LUAD cell lines. **A** qRT-PCR analysis of KCNK3 expression was conducted after KCNK3 overexpression lentivirus transfection in H1975 cells and H1299 cells. **B**, **C** Relative KCNK3 protein expression was measured by western blot after transfection of KCNK3 overexpression lentivirus in H1975 cells and H1299 cells, with results normalized relative to the expression of GAPDH. **D** The effect of KCNK3 on LUAD cell viabilities was detected by CCK-8 assay and expressed as OD values. **E**, **F** EdU-594 staining assay was performed to evaluate the proliferation of KCNK3-overexpressed LUAD cells. The positive ratio was quantified by the counts of EdU-positive cells (red) and total counts of DAPI cells (blue), scale bar 50 µm. **G**, **H** The cell cycle of KCNK3 overexpression LUAD cells was analyzed using flow cytometry. (NC, negative control; OE, overexpression. Data are presented as the mean ± SD of three independent experiments. (*P < 0.05, **P < 0.01, ***P < 0.001).

To explore the effect of KCNK3 on LUAD progression, KCNK3-OE lentiviral plasmid was applied to construct H1975 and H1299 stable transgenic cell lines. qRT-PCR and western blot showed that KCNK3 expression was markedly elevated in KCNK3 virus transfected cells compared with control cells (Fig. 2A–C).

We then adopted the CCK-8 assay to identify the implications of KCNK3 on LUAD proliferative ability, and the result demonstrated that KCNK3 overexpression remarkably inhibited cellular viability in comparison with the control group (*P* < 0.01, Fig. 2D). Additionally, we performed EdU-594 incorporation assay and found that H1299 and H1975 stable cell lines of KCNK3 overexpression presented a lower EdU-594-positive rate in comparison with the corresponding control cells (Fig. 2E, F). Moreover, we validated that elevated KCNK3 could significantly suppress the process of cell cycle and particularly induced cell cycle G0/G1 phase arrest via flow cytometric analysis. (Fig. 2G-H).

To delve further into the biological function of KCNK3, we performed a loss-of-KCNK3-function approach in A549 cells. The construction efficacy was verified through qRT-PCR and western blot. In Figure S1A–C, mRNA and protein expression levels of KCNK3 were downregulated in cells transfected with sh-KCNK3 virus in comparison with sh-NC cells. CCK-8 and EdU-594 assays suggested that the proliferative ability of sh-KCNK3 cells was markedly increased when compared with the control cells (Figure S1D–F). Collectively, the above evidence strongly elucidated that KCNK3 exerted a critical role on LUAD tumorigenesis.

### KCNK3 was involved in the process of glucose metabolism in LUAD cells

**Fig. 3:**
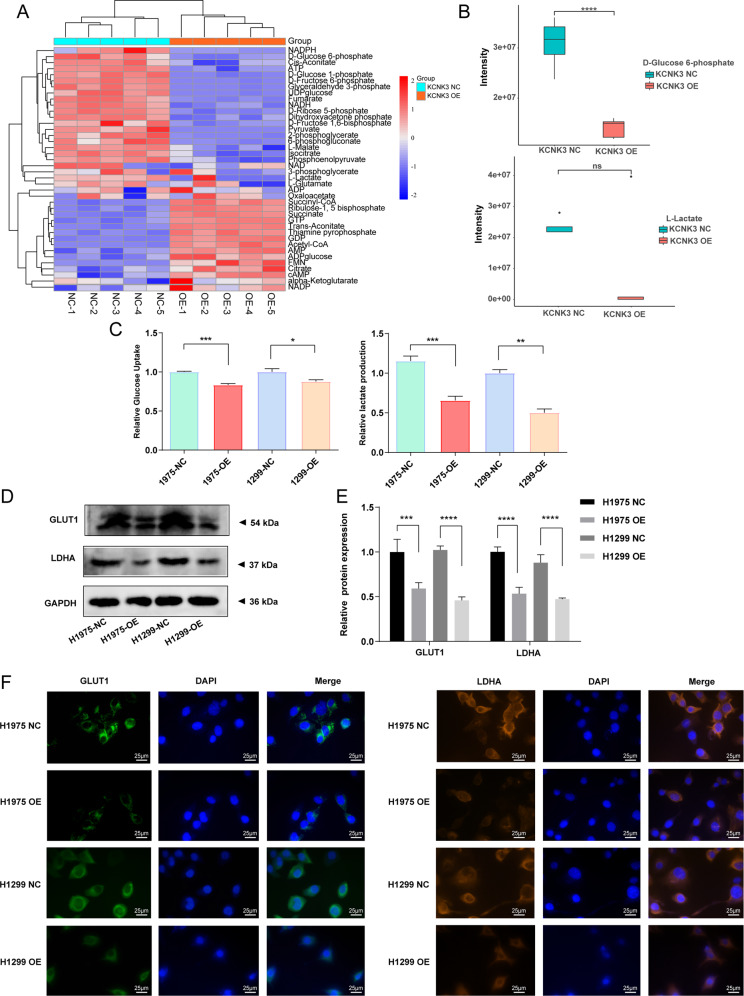
KCNK3 was involved in the process of glucose metabolism in LUAD cells. **A** Heatmap of energy metabolites measured by target metabolomics analysis in KCNK3 overexpression groups and control groups. **B** Metabolic levels of D-glucose 6-phosphate and L-lactate detected by target metabolomics analysis. **C** Relative glucose uptake and lactate production were measured in KCNK3-overexpressed or control LUAD cells. **D**, **E** Western blot analysis of GLUT1 and LDHA protein expressions was performed in KCNK3-overexpressed LUAD cells, with results normalized relative to the expression of GAPDH. **F** GLUT1 and LDHA immunofluorescence was detected to show the glucometabolic alteration in KCNK3-overexpressed LUAD cells, scale bar 25 µm. (NC negative control, OE overexpression. Data are presented as the mean ± SD of three independent experiments. *P < 0.05, **P < 0.01, ***P < 0.001, ****P < 0.0001).

Metabolic abnormality was considered as one of the main driving forces for cancer progression, providing energy and glucose to effectively promote neoplastic proliferation [18–20]. Therefore, we further investigated whether glucose metabolism alteration occurred in KCNK3-mediated proliferation in LUAD cells. We first applied H1299 stable transfection with KCNK3 and control cells to perform a targeted metabolomic analysis. Among the forty energy metabolites identified, a total of 33 glucose metabolites were significantly changed in KCNK3 overexpression group, including decreased D-Fructose 6-phosphate, D-Glucose 6-phosphate, D-Glucose 1-phosphate, and UDP-glucose (Fig. 3A). Although the difference of L-Lactate was not statistically significant between KCNK3-overexpressed cells and control cells, a clear decreased trend was observed (Fig. 3B).

Consequently, we further conducted glucose uptake and lactate production assays to validate whether KCNK3 regulates the glucose metabolism of LUAD cells. Results demonstrated that KCNK3 overexpression induced downregulation of glucose uptake and lactate production, which suggested a decline in cell glucose metabolism (Fig. 3C). Moreover, crucial molecules of glucose metabolism such as glucose transporter 1 (GLUT1) and lactate dehydrogenase A (LDHA) were detected. Western blot results showed that elevated KCNK3 could down-regulate expression levels of GLUT1 and LDHA (Fig. 3D, E). Similarly, GLUT1 and LDHA immunofluorescence experiments in LUAD cells further confirmed the above results (Fig. 3F).

### KCNK3 suppressed the growth and glucose metabolism of LUAD cells in vivo

**Fig. 4:**
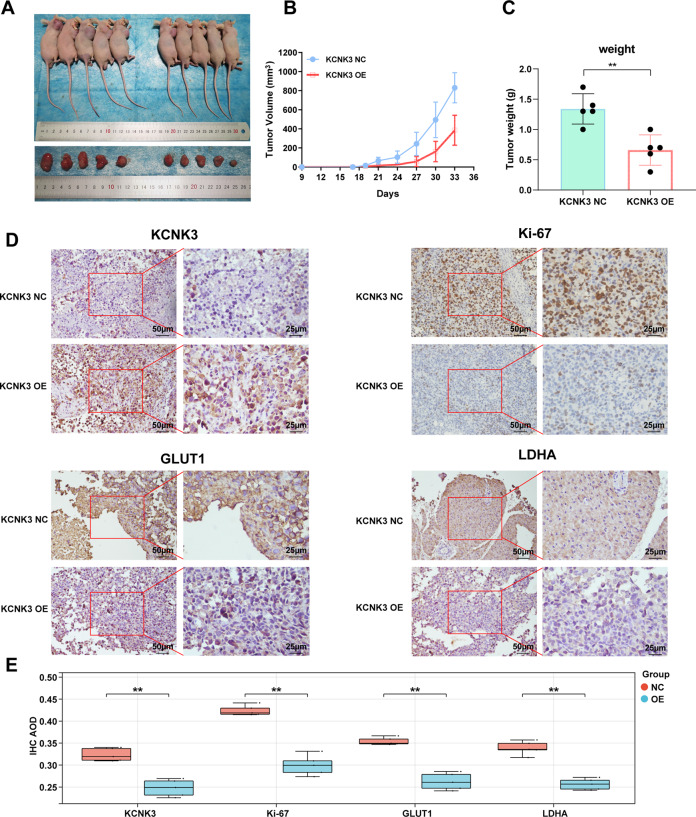
Effect of KCNK3 on the tumor growth and glucose metabolism of LUAD cells in vivo. **A** Representative images of subcutaneous tumors and xenograft tumors was dissected from nude mice (*n* = 5). **B** The growth curves for subcutaneous tumors in nude mice injecting with KCNK3-overexpressed H1299 cells. **C** Comparison of tumor weights from each group of nude mice. **D** The expression of KCNK3, proliferation-associated protein Ki67, and glucometabolic proteins GLUT1 and LDHA in tumor tissues of each group was detected by immunochemistry. Scale bar 50 or 25 µm. (Data are presented as the mean ± SD of three independent experiments. **P < 0.01).

The potential tumorigenicity of KCNK3 on LUAD in vivo was validated in a mouse xenograft tumor model. H1299 KCNK3-overexpressed or control cells (KCNK3-OE and KCNK3-NC, respectively) were subcutaneously injected into BALB/c nude mice. The mice were euthanized 35 days later and tumors were immediately harvested. The result indicated tumors in KCNK3-overexpressed groups were markedly smaller than control groups (Fig. 4A). Additionally, the tumor weight and volume of KCNK3-OE group were remarkedly decreased compared with control group (Fig. 4B, C). Furthermore, immunohistochemical staining was applied to evaluate the expression of KCNK3, proliferation marker Ki67, and the glucose metabolism GLUT1/LDHA in tumor tissue sections. Representative images demonstrated that upregulation of KCNK3 lead to decreased staining intensity of Ki67, GLUT1, and LDHA in the resected tumor when compared with the control group (Fig. 4D).

### KCNK3 activated the AMPK-TXNIP signaling pathway

**Fig. 5:**
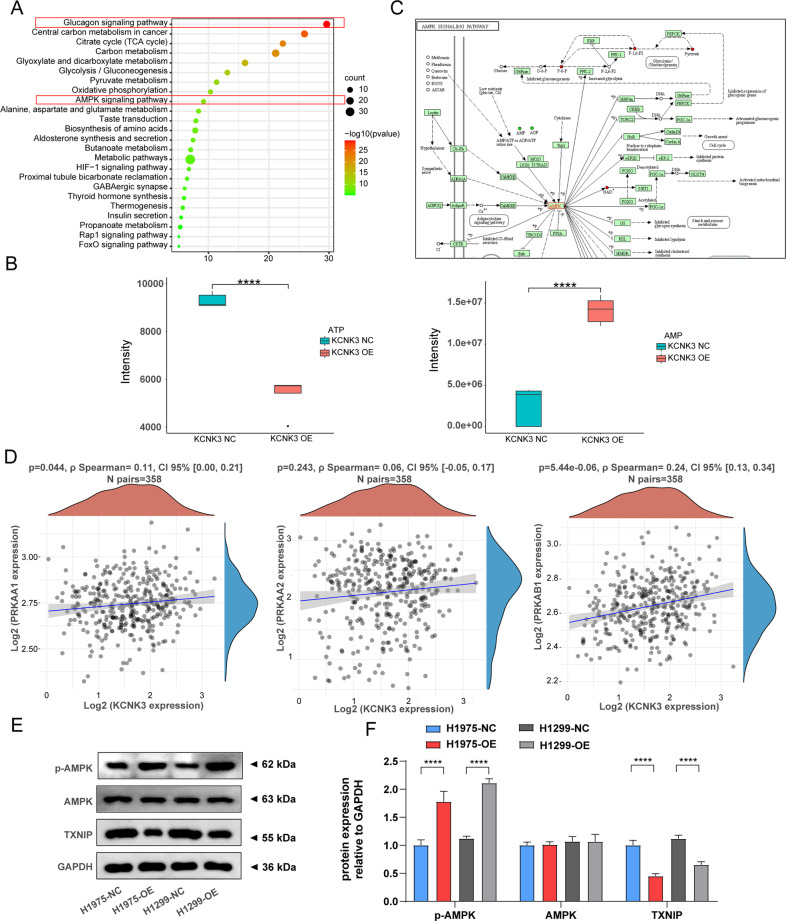
KCNK3 activated the AMPK signaling pathway. **A** Differential metabolites were enriched in the glucagon signaling pathway and AMPK signaling pathway using KEGG databases. **B** Metabolic changes of ATP and AMP were detected by targeted metabolomics analysis. **C** Schematic diagram of the AMPK signaling pathway as presented in the KEGG database (http://www.genome.jp/kegg/). **D** Spearman’s correlation analysis of KCNK3 and AMPK subunits in LUAD patients from TCGA by using clinical bioinformatics database. **E**, **F** Relative p-AMPK, AMPK, TXNIP protein expression was measured by western blot after transfection with KCNK3 lentivirus in H1975 cells and H1299 cells, with results normalized relative to the expression of GAPDH. (Data are presented as the mean ± SD of three independent experiments. ****P < 0.0001).

We further investigated the potential molecular mechanisms underlying how KCNK3 induces cell proliferation and energy metabolism pathways. The targeted metabolomics data were subjected to KEGG enrichment analysis. KEGG pathway analysis indicated that differential metabolites were closely correlated with glucagon signaling pathway and AMPK signaling pathway, which were both involved in the activation of AMPK (Fig. 5A). Previous study has reported AMPK was a key regulator of cellular energy homeostasis that coordinated metabolic processes and was activated by increasing AMP/ATP ratio [21, 22]. Consequently, we further verified whether upregulation of KCNK3 might induce the alteration of ATP and AMP based on metabolomics analysis. In accordance with the above KEGG results, elevated AMP expression accompanied by reduced ATP was observed in KCNK3 overexpression groups when compared with control groups (Fig. 5B), indicating a notable association between KCNK3 and AMPK pathway. In addition to the aforementioned metabolites analyses, we also investigated the biological activities of AMPK from the genetic aspect. Figure 5C revealed AMPK functioned as a central hub of the cellular glucose metabolism network. Moreover, TCGA LUAD dataset was further applied to investigate the correlation between KCNK3 and AMPK. Spearman’s correlation analysis demonstrated that PRKAA1, PRKAA2, and PRKAB1, act as AMPK subunits [23], were positively correlated with KCNK3 expression (Fig. 5D).

Furthermore, the protein expression of AMPK signaling pathway-related molecules were detected by western blot. Our results indicated that the expression levels of phosphorylated AMPK (p-AMPK) were increased whereas thioredoxin-interacting protein (TXNIP) were decreased in KCNK3 overexpression group (Fig. 5E, F). Collectively, we hypothesized that KCNK3 may regulate LUAD cells proliferation and glucose metabolism via AMPK-TXNIP pathway axis.

### KCNK3 inhibited proliferation and glucose metabolism via activation of the AMPK-TXNIP pathway in LUAD cells

**Fig. 6:**
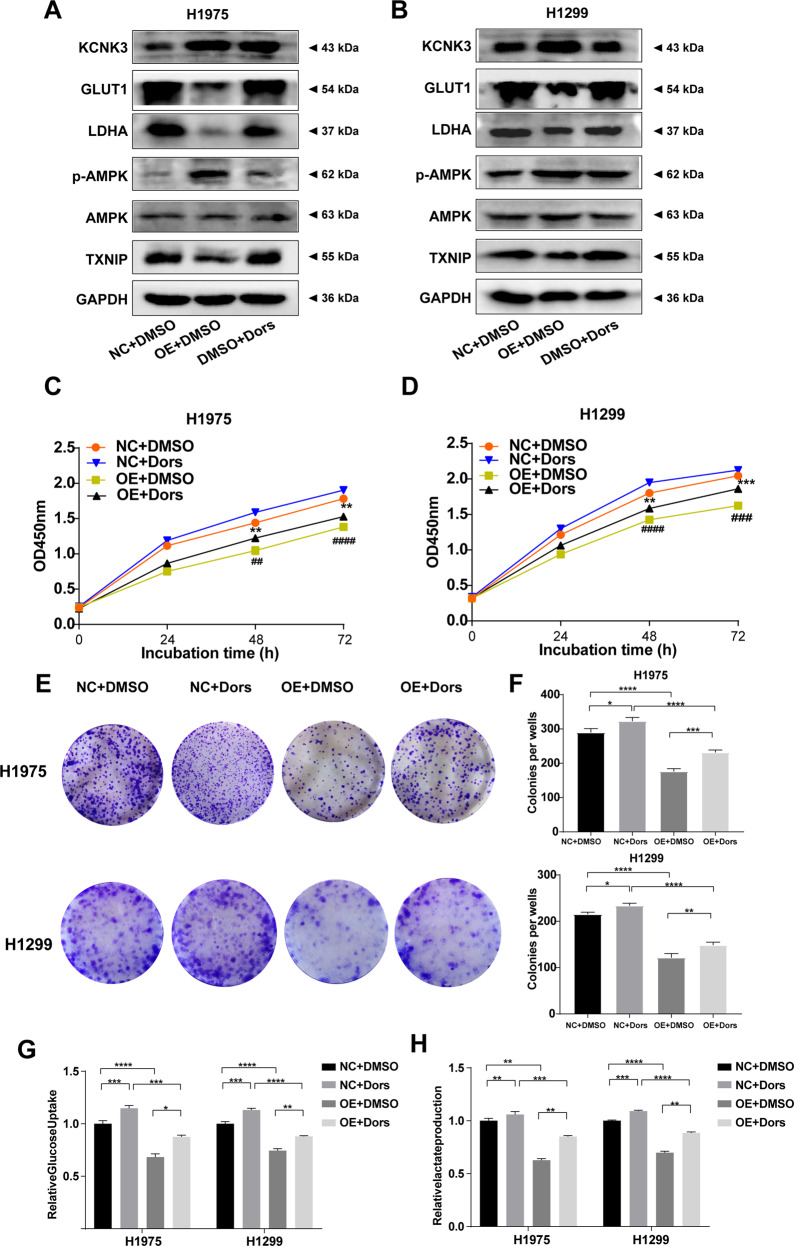
KCNK3 inhibited proliferation and glucose metabolism via activation of the AMPK-TXNIP pathway in LUAD cells. **A**, **B** The expression of KCNK3, GLUT1, LDHA, p-AMPK, AMPK, TXNIP was detected by western blot in LUAD cells after administration with AMPK inhibitor (Dors, Dorsomorphin), with results normalized relative to the expression of GAPDH. **C**, **D** The proliferative curves of LUAD cells were detected by CCK-8 assay and expressed as OD values. **E**, **F** Clone formation assays were performed to evaluate the proliferation of KCNK3 overexpression LUAD cells after treating with AMPK inhibitor. **G**, **H** Relative glucose uptake and lactate production were measured in KCNK3-overexpressed or control LUAD cells after AMPK inhibitor treatment. (Data are presented as the mean ± SD of three independent experiments. *P < 0.05, **P < 0.01, ***P < 0.001, ****P < 0.0001; ^##^*P* < 0.01, ^###^*P* < 0.001, ^####^*P* < 0.0001).

**Fig. 7:**
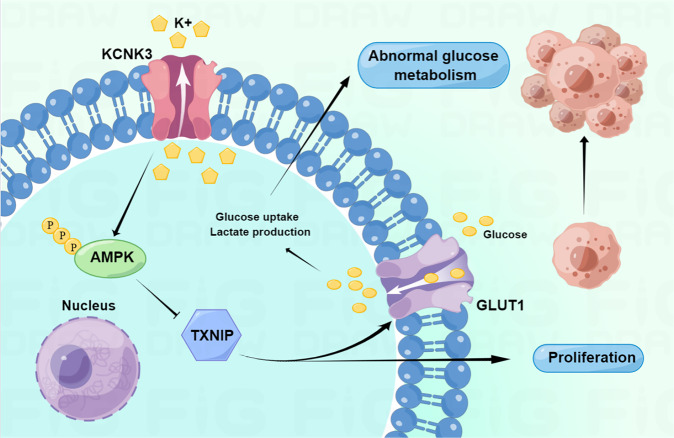
Schematic representation of the proposed mechanism of KCNK3 in LUAD cells. KCNK3 may regulate proliferation and glucose metabolism via AMPK-TXNIP signaling pathway in LUAD cells.

To further explored whether KCNK3-mediated proliferation and energy metabolism through the activation of AMPK pathway, KCNK3-overexpressed H1975 and H1299 cells were treated with an AMPK inhibitor (Dorsomorphin at 5 μM for 48 h). The results presented that the expression levels of KCNK3, GLUT1 and LDHA were restored. Moreover, the expression of p-AMPK and TXNIP was compromised by the AMPK inhibitor in KCNK3 overexpression cells (Fig. 6A, B), which indicated that the proliferative and metabolic potentials of KCNK3 may partially ascribed to the AMPK signaling pathways. Additionally, the consequences of clone formation and CCK-8 assays showed that dorsomorphin can reverse the inhibitory proliferation effect owing to KCNK3 overexpression in H1299 and H1975 stable cell lines (Fig. 6C–F). In addition, glucose uptake and lactate production assay indicated that AMPK pathway inhibitors also reversed the glucose metabolism changes in KCNK3 overexpression cells (Fig. 6G, H). In summary, these results implicated that KCNK3 may modulate AMPK activation and further participate in proliferation and glucose metabolism of LUAD cells. The mechanism diagram of the present study was drawn by Figdraw (www.figdraw.com) and shown in Fig. 7.

## Discussion

Accumulating evidences implicated that K2P channels may be considered as potential therapeutic targets in pain, depression, memory disorders, ischemia/hypoxia and cardiovascular disease [24, 25]. Recently, the critical role of K2P channels in malignant tumors has been increasingly appreciated [26, 27]. In the present study, we confirmed that KCNK3, as one of K2P channels, was remarkably downregulated in LUAD tissues. Moreover, KCNK3 overexpression may inhibit the proliferation and glycometabolism of LUAD cell lines via the activation of AMPK-TXNIP pathway.

In humans, KCNK3 was primarily expressed in lung, brain, pancreas, and placenta [28]. Moreover, it was sensitive to a diverse range of physiological and pharmacological substances such as extracellular pH, unsaturated fatty acids, hypoxia, and intracellular signaling pathways [10, 29]. Previous studies showed that KCNK3 channels were involved in regulations of pulmonary arterial pressure, and aberrant expression of KCNK3 has been determined as a rare factor for idiopathic and familial pulmonary arterial hypertension [30]. Additionally, the regulation of KCNK3 in the proliferative activity has also been reported. For instance, KCNK3 dysfunction could lead to the plasma membrane depolarization of human pulmonary artery smooth muscle cells (hPASMCs) and enhanced the proliferation of hPASMCs [31]. Similarly, Hélène et al. indicated that downregulation of KCNK3 may enhance the proliferation of hPASMCs [31]. The current study also revealed that decreased expression of KCNK3 correlated with poor prognosis in LUAD patients and abnormal KCNK3 expression could regulate the proliferation of LUAD cells including H1299 cells and H1975 cells. However, the specific mechanism by KCNK3 modulated cell proliferation remains further verified.

Glucose metabolism is fundamental for the physiological balance of living organisms [32]. Abnormal glycometabolism is one of the hallmarks of cancer [33].Tumor cells with indefinite proliferation capacity and evolution of a malignant phenotype exhibit a cancer-specific remodeling of glycometabolism [34–36]. Argüello et al. indicated aerobic glycolysis not only supported cellular proliferation but also survival in hypoxic conditions [37]; Pace et al. also reported PKM2, a glycolytic enzyme, induced aerobic glycolysis resulting in tumorigenesis and cancer cell proliferation [38]. Therefore, we performed targeted metabolomics analysis to explore whether glucose metabolism participate in KCNK3-mediated proliferation in LUAD cells. The data demonstrated that there were salient differences in energy metabolites between KCNK3 overexpression group and control group, particularly D-Glucose 1-phosphate and D-Glucose 6-phosphate. Further experiments illustrated that KCNK3 overexpression induced downregulation of glucose uptake and lactate production, as well as the protein expression of GLUT1 and LDHA, indicating that KCNK3 are indeed involved in glucose metabolism in LUAD cells. Consistently, Heller et al. demonstrated that KCNK3 channel participated in regulating glucose homeostasis and insulin secretion [39]. Collectively, the specific mechanism of KCNK3 on glycometabolism regulation was further investigated.

AMPK is the downstream portion of a protein kinase cascade which exhibits an essential role in the modulation of cellular metabolism [40]. Previous study has indicated that AMPK may suppress tumor formation through regulation of cell proliferation, autophagy, and energy metabolism [41]. In addition, AMPK was associated with elevated surface expression and localization of ATP-sensitive K^+^ channel protein [42]. However, as one of the acid-sensitive K2P channels, KCNK3 was rarely reported to interact with AMPK. For studies in HEK 293 cells, TREK-1 and TREK-2 channels as K2P channels could be inhibited by AMPK but not KCNK3 or TASK-3 channels [43], which is not consistent with our study. Our research elucidated that KCNK3 overexpression markedly affected the expression of ATP and AMP through targeted metabolomic analysis. Further analysis of KEGG pathway enrichment revealed that KCNK3-induced differential metabolites were primarily enriched in glucagon signaling pathway and AMPK signaling pathway, suggesting a close functional relationship between KCNK3 and AMPK in LUAD cells. Moreover, western blot results also verified that upregulated KCNK3 can activate the protein expression of p-AMPK. We speculated that the contradictory results may be due to the complexity of signaling pathways in different disease models.

Growing evidences have confirmed that AMPK modulated glucose metabolism by inhibiting TXNIP and consequently induce cellular surface GLUT1 expression [44–46]. Additionally, suppression of AMPK promoted glucose uptake and the production of lactate and pyruvate by elevated expression of the glycolysis-related proteins GLUT1 and LDHA [47]. In the present study, dorsomorphin was applied to inhibit the AMPK biological activity. Dorsomorphin, also known as compound C, a ATP-competitive inhibitor binding to the catalytic energy sensor AMPK [48, 49]. Additionally, dorsomorphin is responsible for suppressing the VEGF type 2 receptor [50] and bone morphogenetic protein (BMP) signaling [51]. While dorsomorphin was frequently considered to inhibit the AMPK pathway. For instance, Yang et al. have reported that dorsomorphin could extensively reduce proliferation rate of colorectal neoplastic cells via targeting AMPK pathways [52]. Our results identified the expression of GLUT1 as well as LDHA was restored, and the expression level of p-AMPK and TXNIP was compromised by the AMPK inhibitor in KCNK3-overexpressed cells, indicating that KCNK3 modulated proliferation and glycometabolism of LUAD cells via activation of the AMPK-TXNIP pathway.

The purpose of the current study was to explore the effects and underlying mechanism of KCNK3 on the tumorigenesis and glucose metabolism of LUAD in vitro and in vivo. We acknowledge, however, that our study has a few notable limitations. Firstly, the number of patients and sample size of clinical specimens were relatively small. Secondly, the clinical prognosis of KCNK3 in LUAD patients was primarily evaluated based on the TCGA database; Therefore, current research lacks some of our own clinical data for prognostic analysis. Thirdly, the roles of AMPK signaling pathway in KCNK3-mediated functions of LUAD have not been validated in vivo. Hence, further studies on accumulate sufficient clinical samples and vivo experiments to verify the above results are warranted.

## Conclusion

In summary, we demonstrated that KCNK3 was markedly downregulated in LUAD cells and predicted a poor clinical prognosis. Moreover, our research verified that KCNK3 acted as a putative tumor suppressor and suppressed LUAD cell proliferation and glucose metabolism by targeting AMPK-TXNIP pathway. Our findings expand a new horizon for the tumorigenesis and glycometabolism of LUAD.

## Materials and methods

### Ethical statement

The study was approved by the Ethics Committee of The Second Affiliated Hospital of Fujian Medical University (approval No. 2020-206) and was performed according to the principles of the Declaration of Helsinki. All participants provided informed written consent. All methods were carried out in accordance with the approved guidelines.

### Cell lines and culture conditions

The following normal and tumor cell lines were applied in this study: human bronchial epithelial cells-HBE, human lung carcinoma cells (A549, H1299, H1975, H460, SPCA-1). All cell lines were obtained from the American Type Culture Collection (ATCC; www.atcc.org). Cells were cultured in DMEM or RPMI-1640 medium containing 10% fetal bovine serum (Invitrogen, USA) and 1% penicillin-streptomycin (Gibco, CA) at 37 °C in the atmosphere containing 5% CO_2_.

### Transcript data analysis

Total RNA of 10 paired stage I LUAD tissues and corresponding normal tissues were extracted using Qiagen RNeasy Mini Kit (Qiagen, Hilden, Germany) following the standard protocol. Then, the maximum residual non-coding RNA was retained after the rRNA was removed from the total RNA. After fragment of rRNA-depleted RNA, the cDNA library was constructed with use of the TruSeq RNA sample Prep Kit (Illumina, USA). mRNA sequencing libraries were prepared following the instruction manual of VAHTS total RNA-seq Library Prep kit for Illumina (Vazyme NR603, China). After completion of the sequencing run, RNA-seq FASTQ files were mapped to the Hg19 reference using STAR, and gene expression was determined using RSEM. Differential expression analysis for mRNA was performed using the DESeq2 R package (https://bioconductor.org/packages/release/bioc/html/DESeq2.html). Differentially expressed genes (DEGs) between two groups were obtained using DESeq2 (v1.10.1). Corrected *P* value of < 0.05 and |Log2 (fold change)| (|Log2 FC|) ≥ 1 were considered statistically significant. Heat maps were generated using hierarchical clustering analysis based on pheatmap R package

### Quantitative real-time polymerase chain reaction

Total RNA from the cell lines and tumor tissues was extracted using TRIzol® Reagent (Invitrogen, USA) according to the manufacturer’s instructions. RNA concentration and quality were quantified by NanoDrop 2000c Spectrophotometer (Thermo Scientific, Fremont, CA, USA). cDNA was synthesized according to the manufacturer’s protocol (Takara, Japan). qRT-PCR was performed using the TB Green PCR kit (Takara, Japan). Real-time PCR reactions were performed using Q5 real-time PCR System (Life Technology, USA). The mRNA expression levels were normalized to GAPDH, and the relative gene expression levels were calculated using the 2^−ΔΔCt^ method. The primers are as follows: KCNK3 forward primer: 5’-TTCTTCCAGGCCTACTACTACT-3’, reverse primer:5’-GTAAGGATGTAGACGAAGCTGA-3’; GAPDH primer: 5ʹ-CACCCACTCCTCCACCTTTG-3ʹ, reverse primer: 5ʹ-CCACCACCCTG TTGCTGTAG-3ʹ.

### CCK-8 assays

CCK-8 assays were performed using the Cell Counting Kit-8 (Beyotime, China). LUAD cell lines were seeded in 96-well plates at a density of 3.0 × 10^3^ cells/well and cultured overnight. After incubation for 24 h, CCK-8 reagents were added and incubated for additional 2 h in the humidified incubator. Absorbance value was measured using a microplate reader at a wavelength of 450 nm.

### 5-Ethynyl-2’-deoxyuridine (EdU) assay

EdU assay was performed using BeyoClick™ EdU Cell Proliferation Kit with Alexa Fluor 594 (Beyotime, China). 2.0 × 10^4^/well cells were added into a 24-well plate and co-cultured with EdU working solution (1:1000) for 2 h at 37 °C in a 5% humidified CO_2_ atmosphere, followed by methanol fixation for 15 min and treatment with 0.5% Triton X-100 for 5 min. Next, cells were incubated with Click reaction solution for 30 min before being stained with Hoechst for 10 min The images were collected with an Olympus microscope (Olympus BX51; Olympus, Japan) and cell counting was conducted by ImageJ software.

### Cell cycle analysis

Flow cytometric analysis was conducted to evaluate the cell cycle. Trypsinized cells were washed with ice-cold phosphate-buffered saline (PBS) and fixed in 70% cold ethanol at 4 °C overnight. overnight. After fixation, cells were incubated with RNase (0.1 mg/mL) for 30 min at 37 °C and stained with propidium iodide (Beyotime, China) for 30 min on ice. Cell cycle analysis was performed by Flow J (version 7.6) software was applied for flow cytometry analysis.

### Lentivirus infection

The lentiviral vector was used to construct stable transgenic LUAD cell lines. H1299, H1975, and A549 cells were seeded into six-well plates at density of 3.0 × 10^5^ cells per well and cultured overnight. When the confluence reached ~20%, cells were infected by KCNK3-overexpressed (OE) and short hairpin (sh) RNA or negative control (NC) lentivirus at a multiplicity of infection of 20 in the presence of 8 μg/ml polybrene. The medium containing virus particles was removed and replaced with complete medium after infection for 16 h. To preserve the property of stable transgenic plants, the presence of 2 μg/ml puromycin (Sigma, St. Louis, MO, USA) were used for following assays.

### Clinical samples and Immunohistochemistry

A total of 58 pairs of LUAD samples and corresponding adjacent tissues were obtained from patients undergoing surgical procedures at The Second Affiliated Hospital of Fujian Medical University. All patients were pathologically diagnosed with lung adenocarcinoma and did not receive any adjuvant radiotherapy and chemotherapy preoperatively. Immunohistochemical staining was performed following a previously described protocol [53]. Paraffin slides (4-mum-thick) were deparaffinized and rehydrated. Subsequently, the sections were boiled in 10 mM citrate buffer (pH 6.0) for 10 min for antigen retrieval, followed by 3% hydrogen peroxide for blocking the endogenous peroxidase activity. Slides were further incubated with the primary antibodies of KCNK3 (A14745, 1:200, ABclonal, China), GLUT1 (ab115730, 1:250, abcam, USA), LDHA (ab47010, 1 µg/ml, abcam, USA) and Ki67 (ab92742, 1:500, abcam, USA) overnight at 4 °C, followed by incubation with horseradish peroxidase (HRP)-conjugated goat anti-rabbit secondary antibody for 30 min. The sections were then stained with peroxidase substrate DAB and counterstained with hematoxylin. Finally, the sections were observed and photographed by Olympus BX51 optimal microscopy.

### Colony formation assay

A density of 5.0 × 10^2^ H1975 and H1299 cells were seeded into six-well plates and maintained in RPMI-1640 medium, respectively. After cultivation for 14 days, the cells were fixed in methanol for 15 min and stained with crystal violet for 20 min. We then calculated the colony formation rate. The assay was conducted three independent times.

### Western blot analysis

Western blot analysis was performed as previously described. Briefly, cells were lysed in RIPA buffer to extract total proteins. Proteins were separated by SDS-PAGE gel and were electrically transferred to a polyvinylidene difluoride membrane. The membrane was blocked with 5% non-fat milk to prevent non-specific binding of antibodies and incubated with specific primary antibodies of KCNK3 (A14745, 1:1000, ABclonal, China), GLUT1 (ab115730, 1:10000, abcam, USA), LDHA (ab47010, 1 µg/ml, abcam, USA), AMPK (ab32047, 1:2000, abcam, USA), p-AMPK (2535 T 1:1000, CST, USA) and TXNIP (ab188865, 1:1000, abcam, USA) at 4 °C overnight. Corresponding HRP-conjugated secondary antibodies were incubated for 1 h at room temperature. Immunoreactive proteins were visualized using Image Quant LAS 4000 (GE Healthcare Life Science, Chicago, IL, USA).

### Cell immunofluorescence

H1975 and H1299 were seeded at 5.0 × 10^3^ cells/well into 24-well plates. Cells were then fixed with 4% paraformaldehyde for 15 min and permeabilized with 0.5% Triton X-100 in PBS for 5 min. Subsequently, cells were incubated with appropriate concentrations of primary antibody of GLUT1 (ab115730, 1:200, abcam, USA) and LDHA (ab47010, 1 µg/ml, abcam, USA) at 4 °C overnight, and then probed with ProteinFind® Goat Anti-Rabbit IgG (H + L), AF488 Conjugate (HS131-01, 1:100, TransGen BioTech, China) for 60 min at room temperature away from light. Next, the secondary antibody solution was removed and the cells were washed thrice by PBS and then incubated with Hoechst for 15 min at room temperature in the dark. Immunofluorescence was visualized and photographed under a fluorescence microscope (OLYMPUS, Tokyo, Japan).

### Tumor xenografts

All animal care and handling procedures were conducted in accordance with the principles of laboratory animal care of the National Institutes of Health (NIH) Guide for the Care and Use of Laboratory Animals, and were approved by The Second Affiliated Hospital of Fujian Medical University. BALB/c nude mice (4 weeks old, male, *n* = 10) were purchased from SLAC Laboratory Animal Co., Ltd (Shanghai, China). Nude mice were maintained in an air-conditioned room with a constant temperature of 22 °C and an alternating 12 h light/12 h dark cycle. Standard laboratory water and food were available ad libitum. The sample size was chosen with adequate power on the basis of the literature and our previous experience [54]. Prior to the experiment, mice were randomly assigned into two different groups (KCNK3-NC or KCNK3-OE). A subcutaneous xenograft tumor model of nude mice was established for in vivo assays. 5.0 × 10^6^ KCNK3-overexpressed H1299 cells or negative control cells were inoculated subcutaneously into right flank of nude mice. We measured and calculated tumor volumes using length × width^2^ × 0.5 every 3 days. Mice were euthanized 33 days after injection. Then xenograft tissues were photographed and immunohistochemical stained.

### Energy metabolism analysis

The metabolites from H1299 cells were extracted using a combination of methanol/acetonitrile/ water (v/v, 2:2:1) under sonication for 1 h in ice baths. The mixture was incubated for 1 h at −20 °C and centrifuged for 20 min at 14,000×*g*, 4 °C, and then transferred to the sampling vial for LC-MS analysis. The significantly different metabolites were determined using a statistically significant threshold of fold change (FC) and two-tailed Student’s *t* tests of the raw data. The *P* value was calculated by one-way ANOVA for multiple groups analysis. Metabolites with FC >1.5 and *P* value <0.05 were considered to be statistically significant metabolites. The identified differential metabolites were used to perform cluster analyses with R package. To identify the perturbed biological pathways, the differential metabolite data were performed KEGG pathway analysis using KEGG database (http://www.kegg.jp).

### Lactate measurement

A Lactate Assay Kit (ab65331, abcam) was used for lactate level measurements. Cell lysates were prepared and then combination of the lactate assay buffer, substrate mix and enzyme mix provided by the kit. The above reaction mix was incubated for 30 min at room temperature. Subsequently, the OD 450 nm of each sample was measured by Spectrophotometer and the concentration of lactate was calculated according to a standard curve.

### Glucose uptake assay

Glucose uptake was detected using Glucose Uptake Assay Kit (ab136955, abcam) following the manufacturer’s protocol. H1975 and H1299 cells were plated in a 96-well plate and starved in serum-free medium overnight to enhance glucose uptake. Glucose starved cells were washed with PBS three times and then stimulated with insulin (±) for 20 min, followed by 2-deoxyglucose (2-DG) for additional 20 min. Subsequently, cells were lysed with lysates buffer, frozen for 15 min at −80 °C and heated for 40 min at 85 °C. The lysates were then neutralized by neutralization buffer and centrifuged after cooling the samples on ice for 5 min, 2-DG-6-phosphate (2-DG6P) standard curve was prepared and measured at 412 nm in Spectrophotometer (Thermo Scientific, Fremont, CA, USA).

### Statistical analysis

Independent experiments were performed and repeated in triplicate. All data were presented as mean ± standard deviation (SD). Statistical significance were analyzed by student’s *t* test with Welch’s correction, one-way ANOVA with Tukey’s posttest where appropriate. In addition, two-way ANOVA followed by Bonferroni’s multiple mean comparisons were applied to analyze group differences. *P* < 0.05 was considered as statistical significance. GraphPad Prism software and SPSS version 22.0 (IBM, USA) were used for analyses.

## Supplementary information

original data.

Supplementary file-Figure S1

H1975-STR Report

H1299-STR Report

A549-STR Report

## Data Availability

All data generated or analyzed in the present study are included in this published article, and its additional information files.
