# Supplementary material for: KCNK3 inhibits proliferation and glucose metabolism of lung adenocarcinoma via activation of AMPK-TXNIP pathway
Source: Cell Death Discov. 2022 Aug 13;8:360. doi: 10.1038/s41420-022-01152-9 (PMC9376064; doi:10.1038/s41420-022-01152-9)
Supplement: Supplementary file 1 — original data. [file 41420_2022_1152_MOESM1_ESM.docx]

Original western blots

Figure 1D

GAPDH (patients 1-3)





KCNK3 (patients 1-3)





GAPDH (patients 4-6 )





KCNK3 (patients 4-6 )





GAPDH (patients 7-9 )





KCNK3 (patients 7-9 )





GAPDH (patients 10-12 )





KCNK3 (patients 10-12 )





Figure 1J

GAPDH-cells





KCNK3-cells





Figure 2B

GAPDH





KCNK3





Figure 3D

GAPDH





GLUT1





LDHA





Figure 5E

GAPDH





p-AMPK





AMPK





TXNIP





Figure 6 A

KCNK3





GLUT1





LDHA





P-AMPK





AMPK








GAPDH





Figure 6B

KCNK3





GLUT1





LDHA





P-AMPK





AMPK





TXNIP





GAPDH





Figure S1B

GAPDH (repeat 1 repeat 2)





KCNK3 (repeat 1 repeat 2)
