# Supplementary material for: KCNK3 inhibits proliferation and glucose metabolism of lung adenocarcinoma via activation of AMPK-TXNIP pathway
Source: Cell Death Discov. 2022 Aug 13;8:360. doi: 10.1038/s41420-022-01152-9 (PMC9376064; doi:10.1038/s41420-022-01152-9)
Supplement: Supplementary file 2 — Supplementary file-Figure S1 [file 41420_2022_1152_MOESM2_ESM.docx]

***Supplementary Material***

**
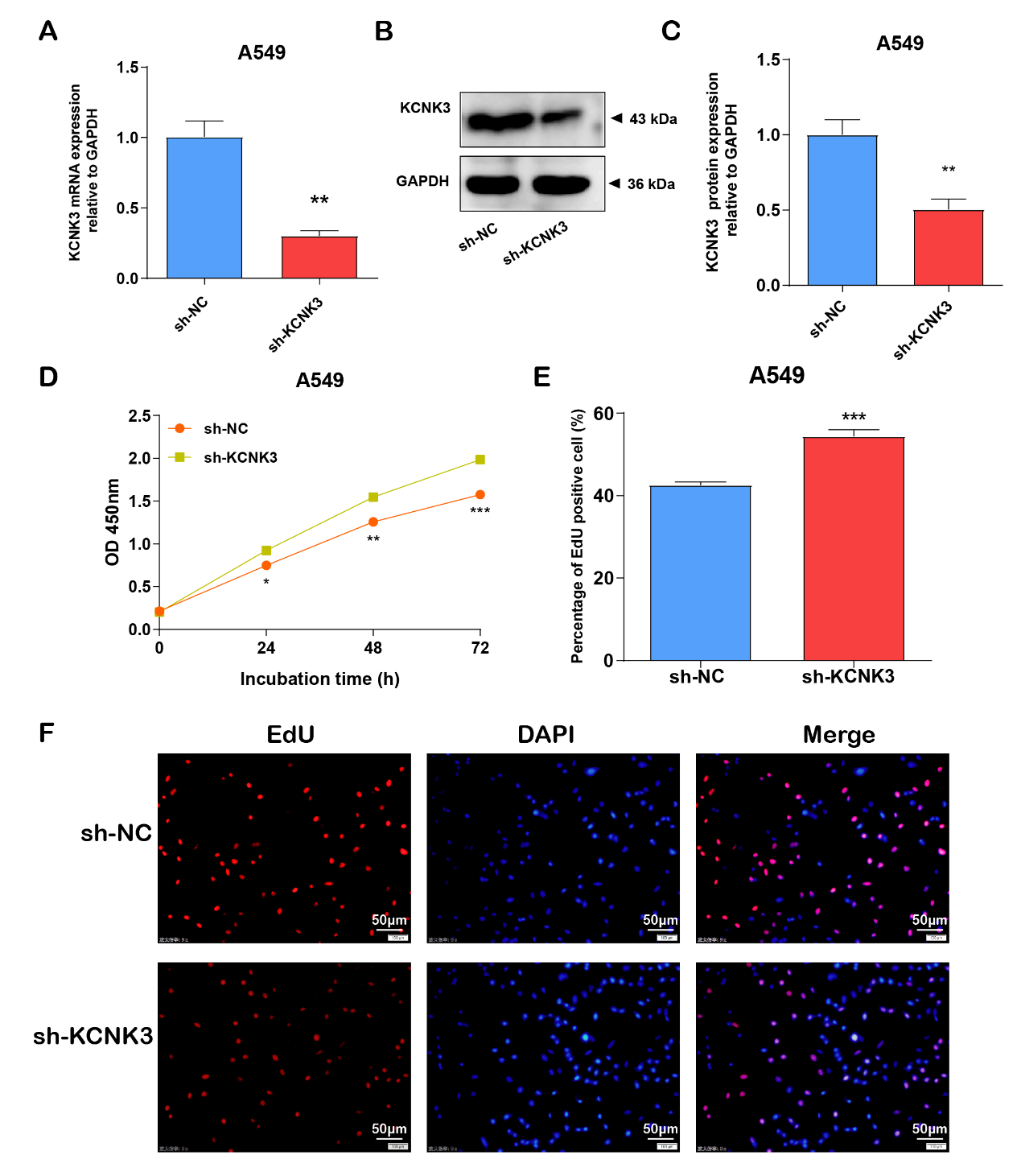
**

**Figure S1**. KCNK3 knockdown promoted the proliferation of LUAD cell lines. (A-C) qRT-PCR and western blot analysis of KCNK3 expression were identified after transfection with KCNK3 knockdown lentivirus in A549 cells. (D) Cell viabilities of LUAD transfected KCNK3 knockdown lentiviruswere detected by CCK-8 assay. (E-F) EdU-594 staining assay was performed to evaluate the proliferation of KCNK3 knockdown LUAD cells. The positive ratio was quantified by the countsof EdU-positive cells (red) and total counts of DAPI cells (blue), scale bar 50 µm. (Data are presented as the mean ± SD of three independent experiments. **P*< 0.05, ***P*< 0.01, ****P*< 0.001).
