# Supplementary material for: KCNK3 inhibits proliferation and glucose metabolism of lung adenocarcinoma via activation of AMPK-TXNIP pathway
Source: Cell Death Discov. 2022 Aug 13;8:360. doi: 10.1038/s41420-022-01152-9 (PMC9376064; doi:10.1038/s41420-022-01152-9)

细胞遗传质量鉴定检测

Cell Line Authentication Service

STR 基因型检测报告

样 品 信 息

样品编号：

客户样本编号	公司编号
105	20180423-01

样品数量： 1

样品性状： 细胞系

检测项目： STR

送检单位： 富衡

检测方法： 用 Axygen 的基因组抽提试剂盒提取 DNA，采用 20- STR 扩增方案扩增，在 ABI 3730XL 型遗传分析仪上对 STR 位点和性别基因 Amelogenin 进行检测。

检 测 结 果

(一)　检验基本情况

	多等位基因	匹配细胞系	细胞库	EV 值	匹配说明
20180423-01	无	NCI-H1975 [H-1975, H1975]	ATCC	0.94	基本匹配

样本基因型检验结果

- 多等位基因指三等位及以上基因现象。
- 本次检测各细胞分型结果良好。

(二)　各样本描述

- 20180423-01：该株细胞 DNA 分型在细胞系检索中找到**基本匹配**的细胞系，ATCC 数据库显示细胞名为 **NCI-H1975 [H-1975, H1975]**，细胞号对应 **CRL-5908**。本次检测在该细胞系中**没有发现多等位基因**。

备注： 待测细胞系与收录于 ATCC, DSMZ, JCRB 和 RIKEN 数据库的细胞系 STR 数据进行比对，未收录于以上细胞库的细胞系将无法匹配。

（三）样本分型结果

细胞 20180423-01 的 STR 位点和 Amelogenin 位点的基因分型结果		
Loci	送检细胞 STR 信息	细胞库细胞 STR 信息

	送检细胞名：105			细胞库细胞名：NCI-H1975 [H-1975, H1975]		
	Allele1	Allele2	Allele3	Allele1	Allele2	Allele3
D5S818	11	12		11	12	
D13S317	10	13		10	13	
D7S820	8	8		8	11	
D16S539	9	12		9	12	
VWA	18	18		18	18	
TH01	7	7		7	7	
AMEL	X	X		X	X	
TPOX	8	11		8	11	
CSF1PO	12	12		12	12	
D12S391	17	17				
FGA	21	24				
D2S1338	17	17				
D21S11	28	28				
D18S51	13	13				
D8S1179	13	13				
D3S1358	14	15				
D6S1043	12	12				
PENTAE	12	12				
D19S433	15	15				
PENTAD	12	13				

其 他 说 明

(一)　分型方案及位点分布

	方案 1	方案 2	方案 3	方案 4
1	TH01	TPOX	D3S1358	AMEL
2	D12S391	VWA	D13S317	D5S818
3	D7S820	D8S1179	D6S1043	D2S1338
4	CSF1PO	PENTAD	D16S539	D21S11
5	FGA		D19S433	D18S51
6	PENTAE			

实验方案及位点

(二)　STR 数据库比对

本公司采用 DSMZ tools 进行细胞系比对，其中包含来自于 ATCC, DSMZ, JCRB 和 RIKEN 数据库的 2455 个细胞系 STR 数据。如果待检测细胞未收录于以上细胞库或这是自行建立的新细胞系将无法进行比对，用户需根据细胞分型结果自行与其他数据库进行比对。

Sample File	Sample Name	Panel	OS	SQ
04_D01_CellLineAuthentication-1-0504.fsa	105	21Plex_STR_Panel_v1.1		

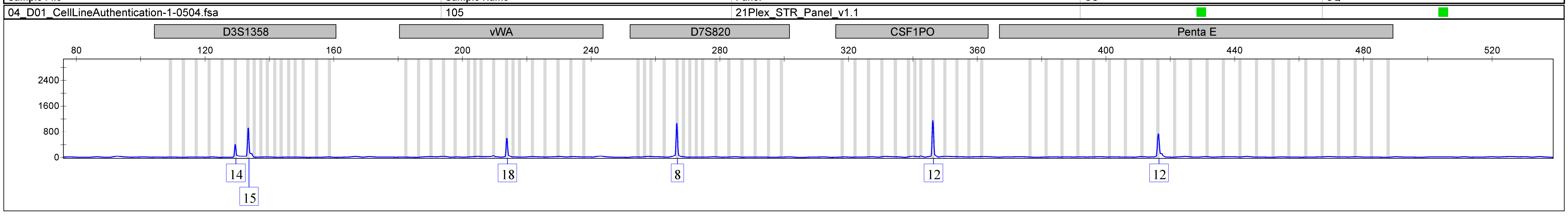

04_D01_CellLineAuthentication-1-0504.fsa | 105 | 21Plex_STR_Panel_v1.1

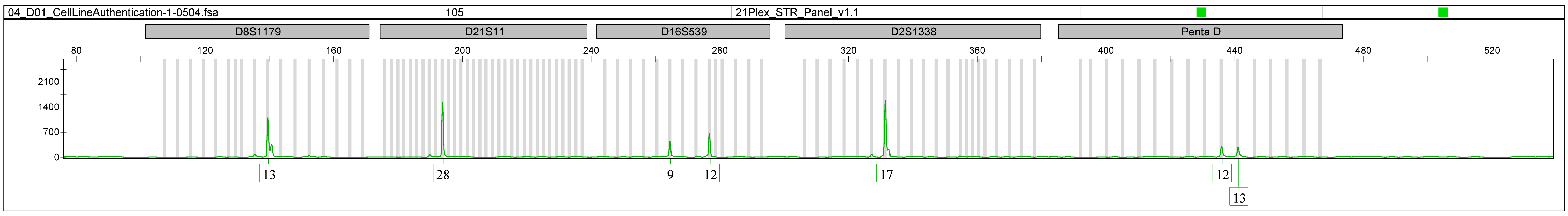

04_D01_CellLineAuthentication-1-0504.fsa | 105 | 21Plex_STR_Panel_v1.1

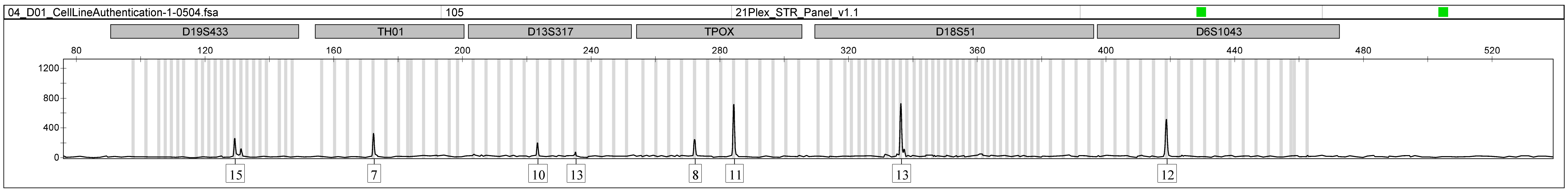

04_D01_CellLineAuthentication-1-0504.fsa | 105 | 21Plex_STR_Panel_v1.1

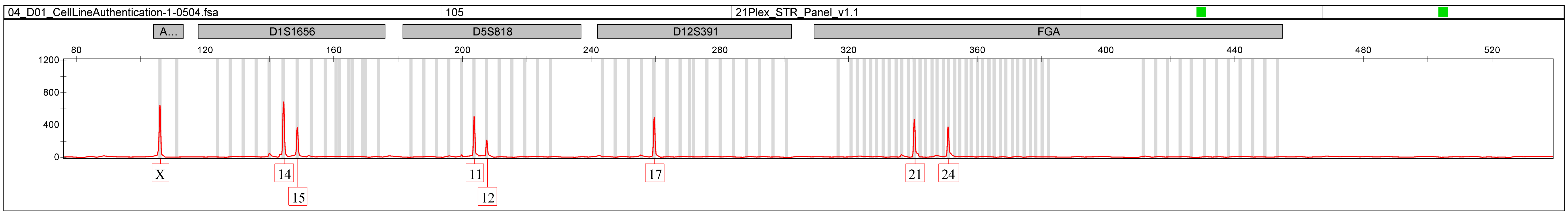

Supplement: Supplementary file 3 — H1975-STR Report [file 41420_2022_1152_MOESM3_ESM.pdf]
